# S190. SCHIZOTYPY & SUICIDALITY: A MENDELIAN RANDOMISATION ANALYSIS

**DOI:** 10.1093/schbul/sby018.977

**Published:** 2018-04-01

**Authors:** Kirstie O’Hare, Richard Linscott, Tony Merriman

**Affiliations:** University of Otago

## Abstract

**Background:**

Subclinical psychotic symptoms, known as schizotypy, predict concurrent and future suicidal ideation and acts. Some suggest this relationship reflects the influence of shared environmental risk factors, or that schizotypy and suicidality are both non-specific indicators of severity of psychopathology. However, this artefactual explanation does not account for the heritability of environmental risk factors, the link between schizotypy and more severe expressions of suicidality, or contemporary theories of suicidality.

**Methods:**

We tested whether schizotypy has a direct (causal) effect on the development of suicidal thoughts using a Mendelian randomisation analysis to avoid problems of reverse causality, confounding, and measurement error associated with traditional observational studies. In Mendelian randomisation analyses, genetic variants are used as a proxy measure for a phenotype in order to make causal inferences about the effect of the phenotype on an outcome. We used a schizophrenia gene risk score (GRS), a measure of schizophrenia liability, as a proxy measure for schizotypy. Participants (n = 4767) were part of the Philadelphia Neurodevelopmental Cohort, a publicly available resource designed to assess behavioural and biological factors contributing to mental illness in young adults (aged 8–21). Regression analyses were used to test relationships.

**Results:**

Schizotypy was found to be a strong predictor of both passive (OR = 1.84, p < .001) and active (OR = 2.69, p < .001) suicidal ideation. No relationship was found between the schizophrenia GRS and schizotypy when analysed in separate ethnic groups to adjust for population stratification (European American; B = .18, p = .708, African American; B = .086, p = .303). No relationship was found between the schizophrenia GRS and passive (OR = .97, p = .721) or active (OR = .99, p = .778) suicidal ideation.

**Discussion:**

The hypothesis that there is a causal relationship between schizotypy and suicidality was not supported, though it is unclear if this is due to the schizophrenia GRS being a poor proxy for schizotypy, or a true absence of a causal relationship. Understanding causal risk factors for suicidality is a key area of research and future research should continue to address this using genetically-sensitive designs.

